# Developing Quality of Medication Error Reports (QoMER): A Consensus-Based Preliminary Framework Using a Modified Nominal Group Technique in the UK

**DOI:** 10.1136/bmjopen-2025-115872

**Published:** 2026-09-02

**Authors:** Manahil Monshi, Zahraa Jalal, Sarah Pontefract, Anthony Cox

**Affiliations:** 1 School of Pharmacy, University of Birmingham, Birmingham, England, UK; 2 Yanbu General Hospital, Yanbu, Al Madinah Province, Saudi Arabia; 3 Pharmacy Department, University Hospitals Birmingham NHS Foundation Trust, Birmingham, UK

**Keywords:** QUALITATIVE RESEARCH, Education, Medical, Quality in health care, Quality Improvement

## Abstract

**Abstract:**

**Objectives:**

To develop a consensus-based preliminary quality assessment framework using a modified nominal group technique (NGT) to describe the key indicators of an optimal medication error report (MER).

**Design:**

A modified NGT consensus study comprising one face-to-face nominal group session and one round of follow-up emails using Microsoft Excel.

**Setting:**

A face-to-face consensus session took place in Birmingham, West Midlands, UK.

**Participants:**

A group of UK national medication safety experts and frontline healthcare professionals, including pharmacists, doctors and nurses (n=11), participated in the nominal group session. Of these, eight were female, and the rest were male. Panel members were selected based on their expertise or practice in medication safety. Non-UK medication safety experts and healthcare professionals were excluded from the study.

**Primary and secondary outcome measures:**

The primary outcome was the development of the proposed Quality of Medication Error Reports (QoMER) framework. Secondary outcomes included the identification of key quality indicators (QIs) for high-quality MERs, inclusion votes, ranking scores and votes used to determine consensus and to inform the exploratory weighted scoring system.

**Results:**

The proposed QoMER scoring framework comprises nine QIs organised under three main themes. The most prioritised QI was that the report should provide sufficient details of the patient and context. Participants ranked the weighted scoring approach as the most appropriate method for combining the completeness and perceived importance of each QI. Qualitative data, including audio-recorded discussions and written ideas, were transcribed and analysed inductively using Braun and Clarke’s thematic analysis. Quantitative data, comprising inclusion votes, ranking scores and weighting values, were summarised descriptively. This integrated analytical approach ensured that both participant discussion and numerical agreement informed the final QoMER scoring framework.

**Conclusions:**

Medication errors lack qualitative depth in reporting, which affects learning, reflection and prevention. QoMER is proposed as a consensus-derived preliminary framework for the structured assessment and categorisation of MER quality by addressing nine QIs. These QIs may help promote more complete and actionable reporting, although further empirical validation is required before conclusions can be drawn about reliability, usability or impact on patient and medication safety.

**STRENGTHS AND LIMITATIONS OF THIS STUDY**

- The study used a structured modified nominal group technique, including silent idea generation, round-robin sharing, clarification, voting and ranking.
- A presurvey, a brief literature review, and a follow-up email were added to support the preparation and confirmation of consensus.
- The face-to-face format enabled real-time clarification but may have introduced conformity or dominance bias.
- The panel included pharmacists, doctors and nurses but was predominantly pharmacy-based and limited to UK participants.
- The weighting system and cut-off points were consensus-derived from a small sample of experts and require future validation and reliability testing.

## Introduction

 Medication errors remain a persistent threat to patient safety worldwide, contributing significantly to preventable harm in different healthcare settings.^1 2^

Robust reporting systems are essential for identifying and mitigating the underlying causes of medication errors. In the UK, the National Reporting and Learning System and, more recently, the Learn from Patient Safety Events (LFPSE) service provide platforms for healthcare professionals to report medication errors.^3^ These systems rely heavily on voluntary incident reports, which, when well-documented, can support local learning and recommendations for safety improvements in healthcare systems.^4^

In 2016, a study analysed over 12 000 primary care safety incident reports. The study revealed that two-thirds of the reports lacked contributing factors or clear explanations for the incidents.^5^ Diagnostic errors, patient assessment issues and vaccination-related incidents were the most prevalent and harmful, particularly among elderly and paediatric patients. Temporal patterns were inconsistent across reports.^5^ The study recommended prioritising these areas in future quality improvement efforts^5^

However, the potential of medication error reports (MERs) is often diminished by inconsistencies in report quality. Reports may omit critical context, present unclear narratives or fail to adequately document underlying factors and patient outcomes. This heterogeneity in quality substantially limits the capacity for meaningful analysis, effective feedback and robust organisational learning.^5^ While various instruments exist to assess the quality of clinical narratives or adverse event documentation, none is specifically tailored to evaluate MERs.^6 7^ There is no standardised tool for benchmarking or enhancing the quality of MERs, which undermines the full learning potential of MER systems.^6 7^

Consensus-building methods are indicated for problems where empirical evidence is limited and expert opinions are needed to translate ideas into guidelines.^8 9^ Structured approaches such as the nominal group technique (NGT) and the Delphi method are widely used in healthcare research to develop guidelines and tools.^10–12^ NGT combines individual idea generation with facilitated discussion and systematic ranking, promoting equal participation and limiting the dominance of individuals’ voices.^10 12^ Unlike Delphi, which requires multiple rounds and is prone to reductions in participant numbers, NGT can achieve agreement within a single meeting and enables real-time clarification of ideas.^11 13^ Given the lack of a gold-standard definition of an optimal-quality MER, the multidisciplinary nature of medication safety, and the need for pragmatic, acceptable quality indicators (QIs), a consensus method using a modified NGT was considered the most appropriate approach to developing a quality scoring framework of MERs.

Although no dedicated quality scoring framework exists for MER, efforts have been made to standardise what information should be captured in patient safety event reports. The WHO’s International Classification for Patient Safety (ICPS) established a conceptual framework and standard terminology for classifying patient safety data.^14^ Similarly, the US Agency for Healthcare Research and Quality (AHRQ) developed the Common Formats for Event Reporting to unify the collection of key data elements across incident reporting systems.^7^ These frameworks specify the key information that should be captured in medication error reports and have informed subsequent efforts to improve the completeness of reported data. For example, Yao *et al*^7^ proposed a ‘Necessary Data Elements Model’ for narrative MERs, identifying the minimal essential information, such as the medication involved, error type, outcome and contributing factors, which should be present to facilitate analysis and learning.^7^ These initiatives underscore the importance of structured reporting; however, they primarily serve as classification standards or reporting guidelines rather than practical tools for assessing the quality of individual MERs.

To remedy this, the present study aimed to develop Quality of Medication Error Reports (QoMER), a consensus-derived preliminary quality assessment framework, to evaluate and enhance the quality of MERs.

## Methods

QoMER is a consensus-derived, preliminary quality assessment framework that describes the key indicators of a high-quality MER. It provides proposed QIs, weights and quality categories to support a structured assessment of report completeness, clarity and actionable usefulness. At this stage of development, QoMER represents an initial framework that requires further empirical testing before it can be implemented in educational curricula or clinical practice.

Our methodology employed a modified NGT. This structured consensus-building approach drew on the expertise of UK-based multidisciplinary professionals to define and refine the QIs for high-quality reports. QoMER is intended to support local quality assurance, steer constructive feedback and ultimately enhance the transformative potential of incident reporting as a cornerstone for improving medication safety.

### Selection of panellists

For successful implementation, an NGT should involve 7 to 12 members.^8 12^ A purposive sampling was conducted to include a panel of 11 medication safety experts and frontline healthcare professionals, including pharmacists, physicians and nurses across England (table 1). Panel members were selected based on their expertise or practice in medication safety, either by experts in England or through LinkedIn. A PhD candidate in Pharmacy Practice facilitated the nominal group session. A total of 45 invitations for participation, covering a range of backgrounds and experiences, were sent via email in April, May and June 2024. Email reminders were sent a week after the initial invitation if the participant did not respond. When the participant accepted the invitation by completing a consent form, another email would follow, containing a link to Microsoft Forms to collect their initial ideas for QIs to describe an optimal MER. The form also facilitated their travel, accommodation and any dietary requirements. Reminder emails were sent to all 11 participants 1 week and 1 day before the nominal group session, providing details on the session location, date and time. This approach was taken to ensure that all participants were well-informed and prepared for the upcoming session.

**Table 1:** Participant characteristics

Number	Occupation/organisation	Sex
1	Professor in pharmacy, university	M
2	Paediatric intensive care nurse, secondary care	F
3	Chief pharmacist, secondary care	F
4	Medication safety officer, secondary care	F
5	Medicine safety consultant pharmacist, secondary care	M
6	Professor in pharmacy, university	F
7	Advanced clinical pharmacist, secondary care	M
8	Assistant professor in nursing, university	F
9	Deputy chief pharmacist and medication safety officer, secondary care	F
10	Lecturer in clinical pharmacy, university	F
11	General practitioner, primary care	F

### Preparatory research

To prepare for this NGT, a thorough literature review was conducted. The literature review included the NGT method, its stages, variations and applications in healthcare research.^8 12^ During the invitation phase of the NGT, participants (n=11) were asked to provide their consent forms and propose QIs to describe an optimal MER (n=8) via Microsoft Forms. The moderator (MM) presented the participants’ ideas and the prior literature on weaknesses in safety incident reports during the second round-robin.

### Assessing consensus

An NGT, also known as an expert panel,^8^ was employed as a consensus method to assess a wide range of QIs. Based on these indicators, the experts determined a quality assessment framework (and therefore a score) for an optimal MER. The conventional NGT, as developed by Delbecq and Van de Ven, included four stages: silent generation, round-robin, clarification and both voting and ranking.^10^ A modified NGT was adopted for this study. In addition to the conventional NGT discussion, the process incorporated three key components: (1) a presurvey to collect preliminary participant input, (2) a presentation of relevant literature on recommendations on quality improvement initiatives in MER and (3) a follow-up email to confirm and finalise consensus on the identified NGT outcomes. This modification enabled participants to familiarise themselves with others’ ideas and to assess the relative importance of those ideas.^15^ The study was piloted with two academics, leading to more concise research questions. Anonymity was not applied because the NGT session was face-to-face, and the moderator only organised and managed the process.

### Data collection

On the day of the nominal group session, the research team asked each participant to introduce themselves by name, current position and organisation to encourage discussion and collaboration. Then, a brief presentation was shown to all participants, explaining the aim, objectives and outcomes of this nominal group session. This presentation communicated the purpose of the NG session, the scope of the topic and the process to be followed.

A face-to-face nominal group session was held in June 2024 at the University of Birmingham, Birmingham, England, moderated by one of the researchers. The session was facilitated through Microsoft Forms and lasted 6 hours with a 1-hour break at midday.

Two main questions were explored: Q1: How would you describe an optimal MER? Q2: What scoring system for these QIs would be appropriate for evaluating MERs? The commonly used four stages of the NGT were employed for each question. Starting with the silent generation of ideas in writing using a pen and a pad provided for each participant, to allow participants to independently generate ideas, issues or recommendations without influence from others. Then, ideas were collected in a terse phrase from each participant in a round-robin fashion, so each participant’s contributions are heard equally, while compiling a comprehensive list of items for discussion without judgement or critique at this stage. Microsoft Forms’ real-time functionality enabled participants to view all posted ideas and avoid duplicates. These two stages were repeated three times until the ideas reached saturation. The third stage, clarifying the ideas, occurred when the moderator read each idea out loud on the board. The participants were given time to discuss, clarify, group similar ideas and eliminate repetitive ones. The fourth stage involved individual voting on which QIs to include or exclude from the quality score and rating them from most to least important. According to previous literature, an idea should receive consensus agreement from 70% or more of participants to be included.^16^ Then, the moderator asked all participants to rate the ideas by dragging and dropping them in order of importance. These responses were aggregated into a horizontal bar chart for each indicator. The purpose of this stage is to collectively rank the most important ideas, creating a prioritised consensus list to inform the quality score of describing an optimal MER.

To finalise the recommendations from this nominal group session, the research team sent an email to all participants. The email required three actions using Microsoft Excel:

1. To approve the rewording of the QIs or suggest another rewording.
2. To indicate a weight out of 5 for each QI as part of the scoring system.
3. To suggest a cut-off point for optimal, adequate and poor MERs out of 100%.

### Data analysis

The session was audio recorded using a digital, encrypted audio recording device. The primary purpose of the recording is to track the number of ideas, stages and duration of the nominal group, facilitating data analysis. In the present study, the collected data were analysed in inductive thematic and quantitative analyses. The inductive thematic analysis was conducted using NVivo V.14 software, following the six-step process described by Braun and Clarke in 2006.^17^ An inductive thematic analysis was used to interpret the rationale underpinning participants’ choices of the final QIs. Initial coding focused on participants’ explanations of why particular reporting details were considered important, how incomplete or vague reports limit learning, and how practical and cultural factors may influence reporting quality. Codes were then reviewed and grouped into candidate themes that explained the conceptual meaning of MER quality. The final themes were checked against both the transcript and the final QIs to ensure they captured the underlying reasoning behind the consensus outcomes.

Quantitative analysis used Microsoft Forms to analyse the ranking stage. The participants’ rankings of the QIs were summed, with higher scores indicating greater importance. Since the first rank gets 10 points, the second rank gets 9 points, and the last rank gets 1 point. Given the small number of participants in the follow-up email (n=10), the weighting values and cut-off point suggestions were analysed descriptively. For each QI weight, the mean was calculated for use in the final weighted scoring system, and distribution was summarised using SD, median, IQR and range. Suggested cut-off points for the MER quality categories were also summarised using mean, SD, median, IQR and range. These statistics were used to describe the degree of variation in expert judgement.

The scoring structure and categorisation cut-off points were developed as pragmatic, consensus-derived decisions rather than psychometrically validated parameters. The 0–3–5 completeness-scoring structure was selected to distinguish three levels of reporting quality: absence of the indicator, partial documentation and complete documentation. This approach was considered more informative than a binary yes/no assessment because it recognised partial reporting while avoiding the potential overprecision of a more granular scale at this early stage of framework development. Using a maximum score of 5 also aligned the completeness scoring with the 1–5 importance-weighting scale.

The weighting methodology was based on expert judgement of the relative importance of each QI. The mean weight for each QI was used to retain the contribution of all participant responses and to produce a proportional weighted score that reflected both completeness and perceived importance. Given the small expert sample and the developmental nature of the study, these weights were summarised descriptively rather than treated as statistically precise estimates. Similarly, the proposed cut-off percentages for classifying MERs as optimal, adequate, poor or incomplete were derived from participants’ suggested thresholds and summarised descriptively. These categories should, therefore, be interpreted as preliminary consensus-derived classifications that require future empirical testing.

### Participation

The NGT session was conducted over a full working day, 7 hours, with participants provided with lunch and refreshments. Participants travelling from outside Birmingham were eligible for reimbursement of travel costs and provision of one night’s accommodation before the session.

#### Results

Eleven medication safety experts and frontline healthcare professionals participated in a single face-to-face nominal group session (participation rate, 24%). Participants represented various healthcare professions and academic backgrounds, including pharmacists, doctors and nurses from across England, primarily Birmingham. Most participants had a pharmacy background (72%), and the majority represented a healthcare organisation (72%). The participants were presented with two research questions.

The first question: How would you describe an optimal MER?

**Stage 1: silent generation:**

**Stage 2: round-robin format recording:** three rounds were repeated, generating 32 ideas, which are listed in online supplemental file A.

**Stage 3: discussion and clarification:** the participants included 13 ideas of QIs. The ideas that received fewer than seven votes were excluded, as shown in online supplemental file B.

**Stage 4: ranking:** the 10 QIs were ranked from most to least important.

**Follow-up email:** the QIs were rephrased and grouped as suggested by the participants. The finalised outcomes of the ranking stage and follow-up email are presented in box 1.

Box 1 **The proposed Quality of Medication Error Reports (QoMER) Framework**

1. The report provides sufficient details of the patient and context:
  - Age.
  - Ethnicity.
  - Sex.
  - Weight (units used in the institution).
  - Location.
  - Diagnosis.
  - Comorbidities.
  - Medication regimen.
  - Medication involved in the incident.
  - (Systems or equipment involved (eg, pump).
2. The report describes the contributing factors/ the circumstances of the error.
3. The report provides sufficient information to categorise the error: error occurrence and patient harm.
4. The report specifies
  - Fact—the actual harm.
  - Opinion—the potential harm.

1. The report provides a complete timeline (date and time) starting from the occurrence of the error and intervention and ending with the report.
2. The report explains the immediate actions taken.
3. The report specifies the stage of the medication process where you feel the error occurred.
  - Stages of the medication process:
  - Prescribing.
  - Transcribing and documenting.
  - Dispensing.
  - Administering. (5Rights: patient, drug, dose, time and route)
  - Monitoring.
4. The report names the reporting healthcare professional and specifies their role.
5. The report explains events, leading to the discovery of the error.

The second question: What scoring system for these QIs would be appropriate for evaluating MERs?

**Stage 1: silent generation**

**Stage 2: round-robin format recording:** one round generated 11 ideas for scoring systems presented in online supplemental file C.

**Stage 3: discussion and clarification:** the participants included four ideas for scoring systems. See table 2.

**Table 2:** The outcomes of stages 3 and 4 of the second question

Ranking	Scoring systems	Inclusion votes
1	Weighted scoring system	11
2	3-point Likert scale (not at all, partially, fully)	10
3	5-point Likert scale	11
4	Simple yes/no system (yes: included, no: not included)	10

**Stage 4: ranking:** participants ranked the scoring system ideas from the most to least appropriate. Then, all participants agreed on the weighted scoring system during the NGT session, as shown in table 2.

**Follow-up email:**

Rephrasing the QIs, detailing the weighted scoring system and outlining the cut-off points for MER quality were the outcomes of the follow-up email.

See online supplemental file D for a detailed spreadsheet of the weighted scoring system.

### Rephrasing of the QIs

Of the 11 participants, 10 responded to the follow-up email by completing the Excel spreadsheet. Most participants concurred with the wording proposed during the NGT session, although some suggested minor modifications.

For QI 1, two participants recommended removing the term ‘race’ and retaining only ‘ethnicity’. Regarding QI 2, two participants proposed removing the word explicitly, while three others suggested merging QIs 2 and 3 into a single, unified indicator. Additionally, four participants emphasised the need for QI 8 to outline the stages of the medication process. In contrast, the revised wording for the remaining QIs was accepted as proposed by the research team.

### Weighted scoring system

The MER would be evaluated against the nine QIs. Completeness of each QI in the report would be assessed out of five, where 0=not present, 3=partially complete and 5=complete. Each participant assigned a weight to each QI based on its importance, with 1=less important, 3=somewhat necessary and 5=most important. The mean weight of each QI was calculated across all the participants. The total weighted score was summed. The total weighted score provided a preliminary estimate of MER quality by combining the completeness of each QI with its consensus-derived importance weight. This score should be treated as an exploratory estimate rather than a validated measure of report quality (online supplemental file D: detailed spreadsheet of the weighted scoring system). The mean weights and distribution measures for each QI are presented in table 3. The highest mean weight was assigned to QI 1; the report provides sufficient details of the patient and context (mean=4.80, SD=0.42; median=5.00, IQR=5.00–5.00; range=4–5), indicating strong agreement among participants regarding its importance. QI 2 also received a high mean weight (mean 4.50, SD 0.85; median 5.00, IQR 4.25–5.00; range 3–5). Greater variation was observed for some other weighted indicators, particularly QI 8, the report names the reporting healthcare professional and specifies their role (mean=3.20, SD=1.69; median=3.50, IQR=1.50–4.75; range=1–5), suggesting less agreement regarding its importance.

**Table 3:** Distribution measures for QI weights

QI	Mean weight	SD	Median	IQR	Range
QI 1	4.80	0.42	5.00	4.00**–**5.00	4-5
QI 2	4.55	0.76	5.00	4.25**–**5.00	3-5
QI 3	3.50	1.18	3.50	3.00**–**4.00	1-5
QI 4	3.20	1.23	3.00	3.00**–**3.75	1-5
QI 5	2.90	0.88	3.00	3.00**–**3.00	1-4
QI 6	3.50	1.27	3.00	3.00**–**4.75	1-5
QI 7	3.30	1.49	3.00	3.00**–**4.75	1-5
QI 8	3.20	1.69	3.50	1.50**–**4.75	1-5
QI 9	2.90	1.37	3.00	2.25**–**3.00	1-5

QI, quality indicator.

### Cut-off points for MERs out of 100%

Finally, the participants were asked to suggest a cut-off percentage to differentiate optimal, adequate, poor and incomplete MERs. The research team calculated the mean percentage for each cut-off and summarised variation using SD, median, IQR and range. An optimal MER must score 83% or more, an adequate report scores 83%–60%, a poor 60%–51% and an incomplete MER would score less than 51%. The distribution measures for the suggested cut-off points are presented in table 4.

**Table 4:** Distribution measures for suggested cut-off points for MER quality

Cut-off point	Mean %	SD	Median	IQR	Range
Optimal MER	83	9.49	80	80.00**–**80.00	70**–**100
Adequate MER	60	12.47	60	50.00**–**60.00	50**–**90
Poor MER	51	15.96	50	42.25**–**50.75	30**–**90

MER, medication error report.

The optimal MER showed a mean of 83% (SD 9.49; median 80%, IQR 80.00–80.00; range 70–100). The adequate MER had a mean of 60% (SD 12.47; median 60%, IQR 50.00–60.00; range 50–90). The poor MER had a mean of 51% (SD 15.96; median 50.00%, IQR 42.25–50.75; range 30–90). These values indicate that participants broadly agreed on the optimal cut-off point, while there was greater variation in suggested lower cut-off points, particularly for poor MERs.

### Thematic qualitative analysis

**Figure 1:**
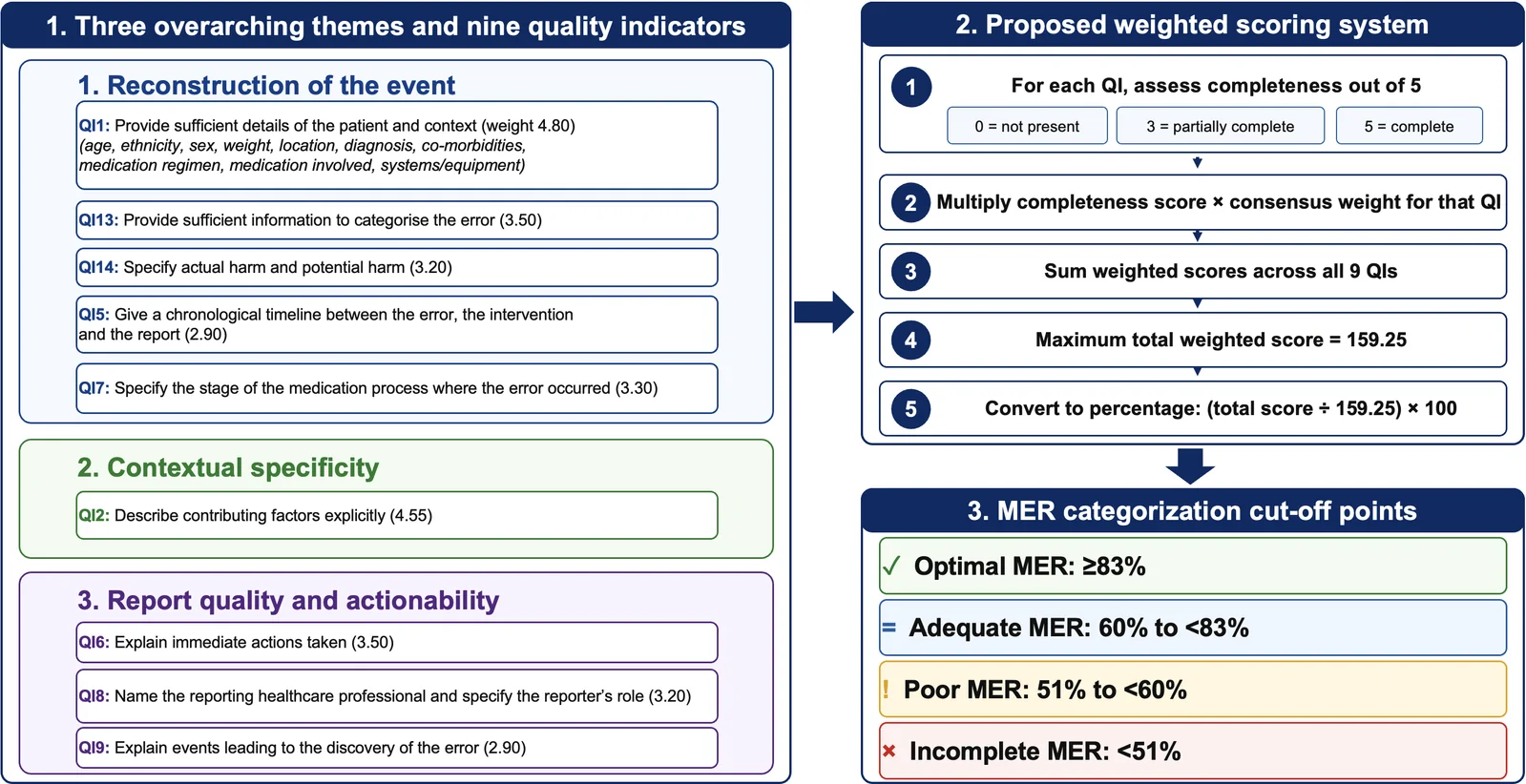
Conceptual figure of the QoMER development. This figure summarises the three overarching themes, nine quality indicators, the proposed weighted scoring system and MER categorisation cut-off points. MER, medication error report; QI, quality indicator; QoMER, Quality of Medication Error Reports.

The research team analysed the session’s raw data from the recorded transcript, along with the final nine QIs (see figure 1). The thematic analysis provided insight into how participants conceptualised a high-quality MER and why particular QIs were prioritised. The themes reflected participants’ underlying reasoning about what makes a report useful for understanding an error, protecting the patient and supporting future learning. Three themes had emerged.

#### Reconstruction of the event

Participants regarded a high-quality MER as one that enables individuals who were not present during the incident to understand what happened, including details about the parties involved, the location, time and stage within the medication-use process. At the beginning of the NGT, the facilitator explained the problem that motivated the study: ‘While all the information is present, the report fails to communicate the whole story, so it affects learning opportunities and prevention of future harm’. This idea was echoed during the discussion when participants clarified that report quality meant whether the reader could grasp the event: ‘If I read that, I understand kind of what’s happened. You’ve got the good information you would need’, while another participant contrasted this with reports where ‘I don’t know what happened’. Participants, therefore, valued factual details such as patient demographics, medication involved, location, timing, stage of the medication-use process and harm because these details allow the event to be reconstructed. However, they also recognised that not all information collected in reporting systems is equally useful. One participant noted that some required fields in incident reporting systems may involve ‘trying to get into patient notes’ for information that is ‘not necessarily relevant’ to medication incidents, and that this may ‘put people off’ completing reports. This suggests that participants were not advocating simply for more data, but for the right information to make the report understandable and useful.

#### Contextual specificity

Participants repeatedly emphasised that contributing factors should be described in a specific and meaningful way. Broad labels such as ‘staffing’ were considered insufficient unless the report explained how they contributed to the medication error. One participant explained that ‘staffing is an issue’, but the actual mechanism may be more specific, such as ‘identical packaging’. Another participant said they wanted to know ‘specifically what went wrong and what the mistake was’.

This theme explains why participants prioritised contextual and contributory factors. They wanted reports to move beyond superficial attribution and provide sufficient detail to support analysis. For example, when discussing how to word the indicator on contributing factors, participants suggested that the report should include the ‘circumstances of the error’ and that these circumstances should allow judgement about the contributory factors. Participants also recognised that the reporter may not always be able to identify all contributory factors, especially if they were not directly involved in the error. One participant noted that the person writing the report may be ‘writing a report about your own error’ or about ‘an error that you’ve picked up’, and in the latter case ‘you might not know what the process was or what those contributing factors were’. This indicates that the final QI on contributing factors reflects a broader conceptual concern: a high-quality MER should support interpretation rather than provide wording to fill the ‘contributing factors’ field. The report should help reviewers distinguish between an immediate description of the incident and the deeper system, environmental, communication, workload, equipment or human factors that may have contributed to it.

#### Report quality and actionability

Participants linked high-quality MERs to actionability. A useful report was one that could support immediate patient safety and help reduce recurrence. One participant stated that the ‘whole purpose of reporting is to analyse errors and reduce the risk of them recurring’, explaining that if a report shows that a handwritten prescription was misread, ‘you can take steps to reduce the recurrence’, whereas if the report ‘doesn’t give you a clue as to how to stop the same error occurring, then it is not a very useful report’. Another participant similarly framed report quality in terms of whether the reader could ‘formulate a way to reduce the future risk of the error recurring and ultimately the risk of patient harm’.

Participants also distinguished between immediate safety actions and longer term learning. One participant described reviewing reports to check whether staff had ‘done everything they need to do to keep that patient safe after that incident’. Another participant explained that after an error, there is both ‘the immediate plan to keep that person safe’ and a second discussion about ‘what happened’, ‘why did that medication error happen’ and ‘what can we put in place… to try and prevent that happening again’. This supports the inclusion of QIs related to immediate actions taken and events leading to the discovery of the error.

Finally, participants discussed the importance of a non-punitive culture. Regarding whether staff names should be included, one participant explained that the issue is ‘confidence that whoever’s made the error will be treated fairly’, because not asking for names may encourage people to report by reducing fear of being ‘picked up’. However, the same participant also noted that knowing the role or level of the person involved may be important for understanding the incident. Another participant emphasised the need to report ‘in a non-judgmental way’, particularly as prescribing roles are becoming more varied across healthcare professionals.

Overall, participants conceptualised an optimal MER as more than a complete form. A high-quality MER should communicate the whole story of the event, provide specific contextual explanations rather than superficial labels, support both immediate patient safety and longer term learning and remain feasible for frontline staff to complete within a fair, non-punitive reporting culture.

## Discussion

This study developed QoMER, a consensus-derived proposed scoring framework comprising nine specific QIs grouped into three overarching themes, to describe an optimal MER. These suggested indicators are intended to support assessment of whether a report communicates the ‘whole story’ of the incident, incorporates contextual specificity and maintains actionability. The proposed indicators, weights and categories may support future work in auditing, training, research and reporting system design; these applications remain potential uses that require empirical evaluation. The highest ranked indicator was the inclusion of sufficient patient and context details in the report, emphasising that patient demographics, clinical setting and medication details are fundamental for understanding errors. This QI aligns with prior literature that identifies core components needed in incident reports to identify learning and inform clinical practice, including a clear description of the incident, contributing factors, classification of the error and severity of harm.^5^ QoMER expands on these by proposing additional elements (such as timeline of events, how the error was detected, the reporter’s role and immediate actions taken) as critical to report quality (box 1). By addressing these nine indicators, our findings underscore the importance of a structured, detailed narrative in MERs and offer a consensus-based description of a high-quality report.

The QoMER scoring framework fills a critical gap in patient safety by proposing a standardised framework to evaluate and benchmark the quality of MERs. Building on the foundational work of Yao *et al*^7^ who developed the Necessary Data Elements Model for assessing the completeness of narrative MERs.^7^ QoMER advances this approach by introducing a weighted scoring system and a proposed categorisation framework that classifies reports as optimal, adequate, poor or incomplete. In addition to evaluating report completeness and clarity, QoMER expands the scope of quality assessment by incorporating key indicators, including comprehensive patient and contextual details, a timeline covering the incident from occurrence to reporting, and documentation of how the error was discovered. By integrating these elements, QoMER offers a more practical, detailed and actionable framework for improving the quality and learning potential of MER, pending empirical validation.

### Global and UK applicability

The QoMER scoring framework may have relevance beyond the UK, as issues such as structural inconsistencies and poor-quality MERs are prevalent in healthcare systems worldwide.^6 18^ A recent international review also highlighted the need for a standardised global framework to ensure consistent analysis of medication errors.^6^ While its principles are broadly applicable, variations in healthcare systems, MER systems, and policies should be considered. By defining consensus-derived QIs, QoMER could serve as a practical template for improving the quality of MERs globally. The shift to the LFPSE service in the UK presents an ideal opportunity to explore whether QoMER indicators could be incorporated into structured MER systems, thereby enhancing data quality and consistency.^3^ On a global scale, QoMER has the potential to improve existing or emerging reporting systems by establishing a clear quality standard; however, its wider adoption will require further validation across diverse international contexts to confirm its reliability and applicability.

### Implications for practice and policy

If empirically validated, the QoMER scoring framework may have practical implications for both frontline healthcare professionals and healthcare administrators. In clinical practice, addressing all QoMER indicators, including patient details, incident timeline, contributory factors and corrective actions in MER systems, as mandatory fields would facilitate complete and timely reporting as well as practical analysis and feedback. Additionally, education and training are crucial for reinforcing high-quality reporting. Recent literature shows that interprofessional education focused on MER enhances the completeness and accuracy of simulated MERs, particularly when healthcare students collaborate across disciplines.^19^ Incorporating QoMER indicators into curricula and training programmes could help foster a culture where MER is recognised not as an administrative task but as a critical learning process that strengthens medication safety.

### Strengths and weaknesses

The NGT is a flexible method and was slightly modified for this research.^16^ The study followed the conventional face-to-face four-phase NGT. It was modified to include a presurvey, a brief literature review, and a follow-up email to support participants in reviewing and confirming their consensus.^15^

To the best of our knowledge, this is the first study to use the NGT method to describe an optimal MER through creating QoMER. Not offering financial incentives to participants implies that they were highly invested in advancing medication safety. At the same time, it was a challenge to find 7–12 participants willing to take a day off from their work and responsibilities and be physically in the same room at the same time. Leading a group of healthcare professionals with senior positions and interpersonal relationships was another challenge. This study sheds light on how to improve medication safety and prevent error recurrence by assessing the quality of MERs.

Since the QoMER scoring framework was developed using 11 UK-based experts, it may limit generalisability. Although the panel was multidisciplinary, perspectives from other countries and healthcare contexts were not addressed. Future research should, therefore, validate whether the nine QoMER indicators are equally relevant across diverse environments.

At this stage, QoMER should be regarded as a proposed framework and developmental tool rather than a validated assessment instrument. The 0–3–5 scoring system, QI weights and cut-off thresholds were derived from expert consensus and descriptive analysis and are, therefore, exploratory. Although distribution measures for QI weights and cut-off point suggestions are reported to improve transparency, these values are based on a small expert sample and should be interpreted descriptively as indicators of consensus variation rather than as measures of statistical precision. Therefore, the usability, feasibility, inter-rater reliability, scoring consistency, validity and appropriateness of the proposed weights and categorisation cut-off points require further evaluation using real-world MERs.

Several methodological limitations should be considered. Although the NGT provided a structured approach, the face-to-face format may have introduced conformity or dominance bias, and the predominantly pharmacy-based panel may have influenced the prioritisation and weighting of indicators in favour of pharmacy-informed perspectives. Additionally, QoMER is a consensus-derived developmental framework that has not yet undergone validation, reliability testing or real-world usability assessment. Its scoring structure, weights and cut-offs are exploratory and require further empirical evaluation across diverse settings and professional groups.

Emerging technologies, including natural language processing (NLP) using transformer models, such as the generative pretrained transformer (GPT), may offer a potential direction for future QoMER development. For example, recent work has explored the use of GPT-based models to extract structured information from free-text MERs, including medications involved, timelines, contributory factors and consequences.^20^ However, the application of such approaches to QoMER remains exploratory. Future research is needed to assess whether NLP methods can reliably identify QoMER indicators, support structured feedback or assist in the quality assessment of MERs. Such work should include empirical validation, manual verification, usability testing and evaluation across diverse reporting systems before any artificial intelligence-assisted application of QoMER could be recommended for routine educational or clinical use. Despite these limitations, QoMER may represent an essential first step towards quantifying report quality and enhancing learning from MERs.

## Conclusion

This tool development study produced QoMER, a consensus-based scoring framework that defines nine key QIs describing an optimal MER and introduces a weighted scoring and categorisation system (optimal, adequate, poor or incomplete). These indicators provide a structured framework for evaluating report quality.

Pending empirical validation, QoMER may provide a structured approach for assessing MER completeness, clarity and actionability and may support future training, audit and reporting system design and could help healthcare organisations strengthen learning from medication errors and foster a culture of transparency and improvement.

By adopting tools like the proposed QoMER, following empirical validation and real-world evaluation, healthcare systems could seek to change reporting from a procedural obligation into a proactive learning mechanism. This could potentially reduce recurring errors and improve medication safety outcomes.

## Supplementary material

10.1136/bmjopen-2025-115872 online supplemental file 1

10.1136/bmjopen-2025-115872 online supplemental file 2

10.1136/bmjopen-2025-115872 online supplemental file 3

10.1136/bmjopen-2025-115872 online supplemental file 4

## Data Availability

All data relevant to the study are included in the article or uploaded as supplementary information.
