# Supplementary material for: Developing Quality of Medication Error Reports (QoMER): A Consensus-Based Preliminary Framework Using a Modified Nominal Group Technique in the UK
Source: BMJ Open. 2026 Sep 2;16(9):e115872. doi: 10.1136/bmjopen-2025-115872 (PMC13548226; doi:10.1136/bmjopen-2025-115872)
Supplement: Supplementary file 1 [file bmjopen-16-9-s004.pdf]

Title: Developing QoMER—Quality of Medication Error Reports: A Consensus-Based Scoring Tool Using a Modified Nominal Group Technique in the UK

Quality of Medication Error Reports (QoMER) Scoring Tool

QI no.	Quality indicators (QIs)	completeness score out of 5	weight of each QI out of 5	Weighted score	
QI 1	QI1:Provide sufficient details of the patient and context:<br>Age<br>Ethnicity<br>Sex<br>Weight (units used in the institution)<br>Location<br>Diagnosis<br>Co-morbidities<br>Medication regimen<br>Medication involved in the incident<br>(Systems or equipment involved (e.g. pump)	5	4.80	24.00	
QI 2	QI2: Describe contributing factors explicitly	5	4.55	22.75	
QI 3	QI3: Provide sufficient information to categorise the error	5	3.50	17.50	
QI 4	QI4: Specify<br>• Fact - The actual harm<br>•Opinion - The potential harm	5	3.20	16.00	
QI 5	QI5: Give a chronological timeline between the error, the intervention and the report	5	2.90	14.50	
QI 6	QI6: Explain immediate actions taken	5	3.50	17.50	
QI 7	QI7: Specify the stage of the medication process where the error occurred	5	3.30	16.50	
QI 8	QI8: Name the reporting healthcare professional<br>•Specify the role of the reporter	5	3.20	16.00	
QI 9	QI9: Explain events leading to the discovery of the error.	5	2.90	14.50	
				32	159.25
			0 = not present	1 = less important QI	5.00
	Suggest a cut-off point of an optimal medication error report (out of 100%)		3 = partially complete	3 = somewhat important QI	100%
	Poor	51	5 = complete	5 = most important QI	
	Adequate	60			
	Optimal	83			
