# Supplementary material for: Developing Quality of Medication Error Reports (QoMER): A Consensus-Based Preliminary Framework Using a Modified Nominal Group Technique in the UK
Source: BMJ Open. 2026 Sep 2;16(9):e115872. doi: 10.1136/bmjopen-2025-115872 (PMC13548226; doi:10.1136/bmjopen-2025-115872)
Supplement: Supplementary file 2 [file bmjopen-2025-115872.reviewer_comments.pdf]

PEER REVIEW HISTORY

BMJ Open publishes all reviews undertaken for accepted manuscripts. Reviewers are asked to complete a checklist review form and are provided with free text boxes to elaborate on their assessment. These free text comments are reproduced below.

ARTICLE DETAILS

Title (Provisional)

Developing QoMER - Quality of Medication Error Reports: A Consensus-Based Preliminary Framework Using a Modified Nominal Group Technique in the UK

Authors

Monshi, Manahil; Jalal, Zahraa; Pontefract, Sarah; Cox, Anthony

---

VERSION 1 - REVIEW

**Reviewer**	**1**
**Name**	**Kelly, James**
**Affiliation**	**University College London**
**Date**	**07-Apr-2026**
**COI**	**None**

Thank you for the opportunity to review this manuscript which addresses a gap in the literature. It would benefit from some minor reframing as a tool development study, with claims about what QoMER enables moderated until empirical validation (inter-rater reliability, pilot testing on real reports) has been conducted. This could be more explicitly acknowledged in limitations. The weighted scoring system would benefit from reporting of dispersion measures for both the QI weights and cut-off points. The thematic analysis could use more explanation and greater depth as it currently reads like restatement of the QI list in thematic form. Also, please resolve the inconsistency between the text (which removes "race" from QI1) and the supplementary spreadsheet (which retains it), and correct some minor typographical errors including "Orgnaization," "Adequet," and "events lead to."

**Reviewer**	**2**
**Name**	**Builes-Montaño, Carlos E.**
**Affiliation**	**Universidad de Antioquia, Internal Medicine**
**Date**	**15-May-2026**
**COI**	**None**

This manuscript addresses an important and underexplored area in medication safety by proposing a consensus-derived framework for evaluating the quality of medication error reports (MERs). The topic is highly relevant to patient safety and incident reporting research, and the use of a modified nominal group technique (NGT) is appropriate for generating expert consensus in the absence of established standards. The manuscript is generally well written, logically structured, and supported by relevant literature. The development of QoMER represents a potentially valuable contribution to efforts aimed at improving the completeness and actionability of medication error reporting.

However, several conceptual and methodological issues should be addressed before the manuscript is suitable for publication.

Major comments

1. Clarification of the conceptual scope and intended purpose of QoMER

The manuscript would benefit from a clearer operational definition of QoMER and its intended use. At present, QoMER is described interchangeably as a scoring tool, quality framework, audit instrument, educational resource, and implementation tool. This creates conceptual ambiguity regarding what the framework is intended to accomplish at its current stage of development.

The authors should clearly define whether QoMER is intended primarily as:

• a preliminary quality assessment framework,

• an audit tool,

• an educational instrument,

• a research tool,

• or a broader implementation framework.

It is recommended that the manuscript explicitly state that QoMER represents a consensus-derived preliminary framework rather than a fully validated measurement instrument.

2. Overstatement of implementation readiness and applicability

Several statements throughout the abstract, discussion, and conclusions imply that QoMER is ready for implementation and capable of improving medication safety outcomes. However, the present study focuses on consensus-based indicator development and does not include validation, reliability testing, usability assessment, or real-world implementation.

Accordingly, claims regarding the ability of QoMER to standardise reporting, improve reporting quality, or enhance patient safety should be moderated. The manuscript should more clearly distinguish between:

• development of the framework,

• and formal validation or implementation.

Phrases such as “may support,” “proposed framework,” or “consensus-derived tool” would be more appropriate than stronger implementation-oriented language.

3. Weighted scoring system and cut-off thresholds require stronger justification

The weighted scoring system and the proposed cut-off categories (“optimal,” “adequate,” “poor,” and “incomplete”) appear largely expert-derived and exploratory. The manuscript currently provides limited methodological justification for:

• the 0–3–5 scoring structure,

• the weighting methodology,

• and the selected threshold percentages.

Additional explanation is recommended regarding the rationale for these scoring decisions. The authors should also explicitly acknowledge that these thresholds are preliminary and require future psychometric validation and empirical testing.

4. Need for stronger emphasis on methodological limitations

The manuscript acknowledges several limitations; however, additional methodological concerns should be discussed more explicitly.

In particular:

• The face-to-face format of the NGT may have introduced conformity or dominance bias despite facilitation efforts.

• The panel composition was predominantly pharmacy-based, which may have influenced prioritisation of specific indicators.

• QoMER has not yet undergone psychometric validation, inter-rater reliability testing, or real-world usability assessment.

• The weighting structure and score thresholds should be described as exploratory and consensus-derived rather than validated.

Strengthening these limitations would improve methodological transparency and align conclusions more closely with the evidence generated by the study.

5. Conceptual figure recommended

The manuscript would benefit substantially from inclusion of a conceptual figure summarising:

• the three overarching domains/themes,

• the nine quality indicators,

• the weighted scoring approach,

• and the final report categorisation framework.

Currently, much of this information is embedded within text and supplementary material, making the conceptual structure of QoMER more difficult to follow.

Minor comments

1. The abstract is generally clear and well structured; however, some wording still overstates the maturity and applicability of QoMER. More cautious language is recommended throughout.

2. The discussion section could be shortened slightly to reduce repetition and improve focus.

3. The section discussing artificial intelligence and NLP applications is interesting but somewhat speculative relative to the scope of the current study. Consider shortening this section or framing it more cautiously as a future research direction.

Overall recommendation

This study addresses a meaningful gap in medication safety research and has clear potential relevance for improving the structure and evaluation of medication error reporting. However, substantial revisions are needed to better calibrate the conclusions, clarify the intended role of QoMER, and align the claims with the developmental nature of the current evidence.

VERSION 1 - AUTHOR RESPONSE

Reviewer: 1
Dr. James Kelly, University College London
Comments to the Author:

RC 1: Thank you for the opportunity to review this manuscript which addresses a gap in the literature. It would benefit from some minor reframing as a tool development study, with claims about what QoMER enables moderated until empirical validation (inter-rater reliability, pilot testing on real reports) has been conducted. This could be more explicitly acknowledged in limitations.

AR1: Thank you for reviewing our manuscript and providing your comments. The manuscript has been updated to frame QoMER as a consensus-based tool development study rather than a validated assessment instrument. We have revised the abstract, discussion, and conclusion to use more moderate statements using terms such as “proposed tool,” “designed to support,” and “pending empirical validation,” about what QoMER currently enables. We have also expanded the limitations section to explicitly state that QoMER has not yet undergone empirical validation, including inter-rater reliability testing or pilot application to real-world medication error reports. Future research will need to assess its usability, reliability, feasibility and validity before wider implementation.

RC2: The weighted scoring system would benefit from reporting of dispersion measures for both the QI weights and cut-off points.

AR2: Thank you for your comment. We acknowledge that reporting mean values only for the QI weights and cut-off points does not adequately reflect the variability in participants' responses. Consequently, the Methods, Results and Strengths and Weaknesses sections have been revised to specify that, due to the limited number of participants in the follow-up round (n=10), the weighting values and cut-off point suggestions were analysed using descriptive methods.

Specifically, we now report distribution measures for each QI weight and the suggested cut-off point, including the standard deviation, median, interquartile range, and range. These values have been added to the Results section to improve transparency regarding the level of agreement and variation in expert judgement.

RC3: The thematic analysis could use more explanation and greater depth as it currently reads like restatement of the QI list in thematic form.

AR3: Thank you for your comment. We have revised the thematic analysis section and discussion using the NGT transcript to provide a deeper insight into the qualitative findings. The analysis was also supported by participants' quotations.

RC4: Also, please resolve the inconsistency between the text (which removes "race" from QI1) and the supplementary spreadsheet (which retains it), and correct some minor typographical errors including "Orgnaization," "Adequet," and "events lead to."

AR4: Thank you for identifying this inconsistency and the typographical errors. We have revised the supplementary D spreadsheet so that QI1 is consistent with the main manuscript: "race" has been removed and "ethnicity" has been retained, in line with the revised QoMER wording presented in Box 1 and the follow-up email results. We have also corrected the typographical errors throughout the manuscript and supplementary materials, including "Orgnaization" to "Organization," "Adequet" to "Adequate," and "events lead to" to "events leading to."

Reviewer: 2
Dr. Carlos E. Builes-Montaño, Universidad de Antioquia, Hospital Pablo Tobón Uribe
Comments to the Author:
This manuscript addresses an important and underexplored area in medication safety by proposing a consensus-derived framework for evaluating the quality of medication error reports (MERs). The topic is highly relevant to patient safety and incident reporting research, and the use of a modified nominal group technique (NGT) is appropriate for generating expert consensus in the absence of established standards. The manuscript is generally well written, logically structured, and supported by relevant literature. The development of QoMER represents a potentially valuable contribution to efforts aimed at improving the completeness and actionability of medication error reporting.
However, several conceptual and methodological issues should be addressed before the manuscript is suitable for publication.

Major comments
RC1: Clarification of the conceptual scope and intended purpose of QoMER
The manuscript would benefit from a clearer operational definition of QoMER and its

intended use. At present, QoMER is described interchangeably as a scoring tool, quality framework, audit instrument, educational resource, and implementation tool. This creates conceptual ambiguity regarding what the framework is intended to accomplish at its current stage of development.
The authors should clearly define whether QoMER is intended primarily as:
- **a preliminary quality assessment framework,**
- **an audit tool,**
- **an educational instrument,**
- **a research tool,**
- **or a broader implementation framework.**

It is recommended that the manuscript explicitly state that QoMER represents a consensus-derived preliminary framework rather than a fully validated measurement instrument.

AR1: Thank you for your comment. We agree that the conceptual scope and intended purpose of QoMER required a clearer definition. In response, we have revised the manuscript to provide an explicit definition of QoMER and to distinguish its current developmental status from its potential future applications.

We now define QoMER as a consensus-derived preliminary quality assessment framework designed to describe the key indicators of an optimal quality medication error report and to propose a structured scoring approach for future empirical validation. We have clarified that, at this stage, QoMER should not be interpreted as a fully validated measurement instrument. Rather, it represents an initial framework developed through expert consensus.

We have also revised the Methods, Discussion, Strengths and Weaknesses, and Conclusion sections to make clear that potential uses of QoMER as an audit tool, educational instrument, research measure or implementation framework remain exploratory and require further validation.

RC2: Overstatement of implementation readiness and applicability
Several statements throughout the abstract, discussion, and conclusions imply that QoMER is ready for implementation and capable of improving medication safety outcomes. However, the present study focuses on consensus-based indicator development and does not include validation, reliability testing, usability assessment, or real-world implementation.
Accordingly, claims regarding the ability of QoMER to standardise reporting, improve reporting quality, or enhance patient safety should be moderated. The manuscript should more clearly distinguish between:
- **development of the framework,**
- **and formal validation or implementation.**

Phrases such as "may support," "proposed framework," or "consensus-derived tool" would be more appropriate than stronger implementation-oriented language.

AR2: Thank you for your comment. We revised the abstract, discussion, strengths and limitations, and conclusion to moderate statements about QoMER's implementation readiness and applicability. Now, QoMER is described as a consensus-derived proposed framework for structured assessment of medication error report quality, not as a validated or implementation-ready tool. We clarified that this study focuses on framework development, and formal validation, reliability testing, usability assessment, pilot testing, and implementation studies

are needed before QoMER can be recommended for routine practice or linked to medication safety improvements.

RC3: Weighted scoring system and cut-off thresholds require stronger justification
The weighted scoring system and the proposed cut-off categories ("optimal," "adequate," "poor," and "incomplete") appear largely expert-derived and exploratory. The manuscript currently provides limited methodological justification for:
• the 0–3–5 scoring structure,
• the weighting methodology,
• and the selected threshold percentages.
Additional explanation is recommended regarding the rationale for these scoring decisions. The authors should also explicitly acknowledge that these thresholds are preliminary and require future psychometric validation and empirical testing.

AR3: Thank you for this important comment. We agree that the weighted scoring system and cut-off thresholds required clearer justification and more cautious interpretation. We have revised the Methods section to explain that the 0–3–5 scoring approach was selected pragmatically to distinguish between absent, partial and complete reporting, while avoiding the limitations of binary scoring and the risk of over-precision at this stage of development. We have also clarified that the weighting approach was based on expert judgement, with mean values used to reflect both completeness and perceived importance.

We have further clarified that the cut-off categories were derived from participant-suggested thresholds and summarised using descriptive statistics. These categories are now explicitly described as preliminary, consensus-based classifications rather than validated thresholds. The Methods, Results and Discussion sections have been revised to emphasise that the scoring system, weights, and cut-off points require future empirical testing using real-world MERs, including evaluation of usability, feasibility, reliability and validity.

RC4: Need for stronger emphasis on methodological limitations
The manuscript acknowledges several limitations; however, additional methodological concerns should be discussed more explicitly.
In particular:
• The face-to-face format of the NGT may have introduced conformity or dominance bias despite facilitation efforts.
• The panel composition was predominantly pharmacy-based, which may have influenced prioritisation of specific indicators.
• QoMER has not yet undergone psychometric validation, inter-rater reliability testing, or real-world usability assessment.
• The weighting structure and score thresholds should be described as exploratory and consensus-derived rather than validated.
Strengthening these limitations would improve methodological transparency and align conclusions more closely with the evidence generated by the study.

AR4: Thank you for your comment. We have strengthened the Strengths and weaknesses section to more clearly acknowledge methodological limitations, including potential conformity or dominance bias in the face-to-face NGT format and the predominantly pharmacy-based panel, which may have influenced indicator prioritisation and weighting. We have also clarified that QoMER is a preliminary, consensus-derived framework that has not yet undergone validation, reliability testing or usability assessment. Additionally, we

emphasise that the scoring structure, weights and cut-off points are exploratory rather than validated.

RC5: Conceptual figure recommended
The manuscript would benefit substantially from inclusion of a conceptual figure summarising:
• the three overarching domains/themes,
• the nine quality indicators,
• the weighted scoring approach,
• and the final report categorisation framework.
Currently, much of this information is embedded within text and supplementary material, making the conceptual structure of QoMER more difficult to follow.

AR5: We appreciate your comment. A conceptual figure illustrating QoMER development has been added to the manuscript.

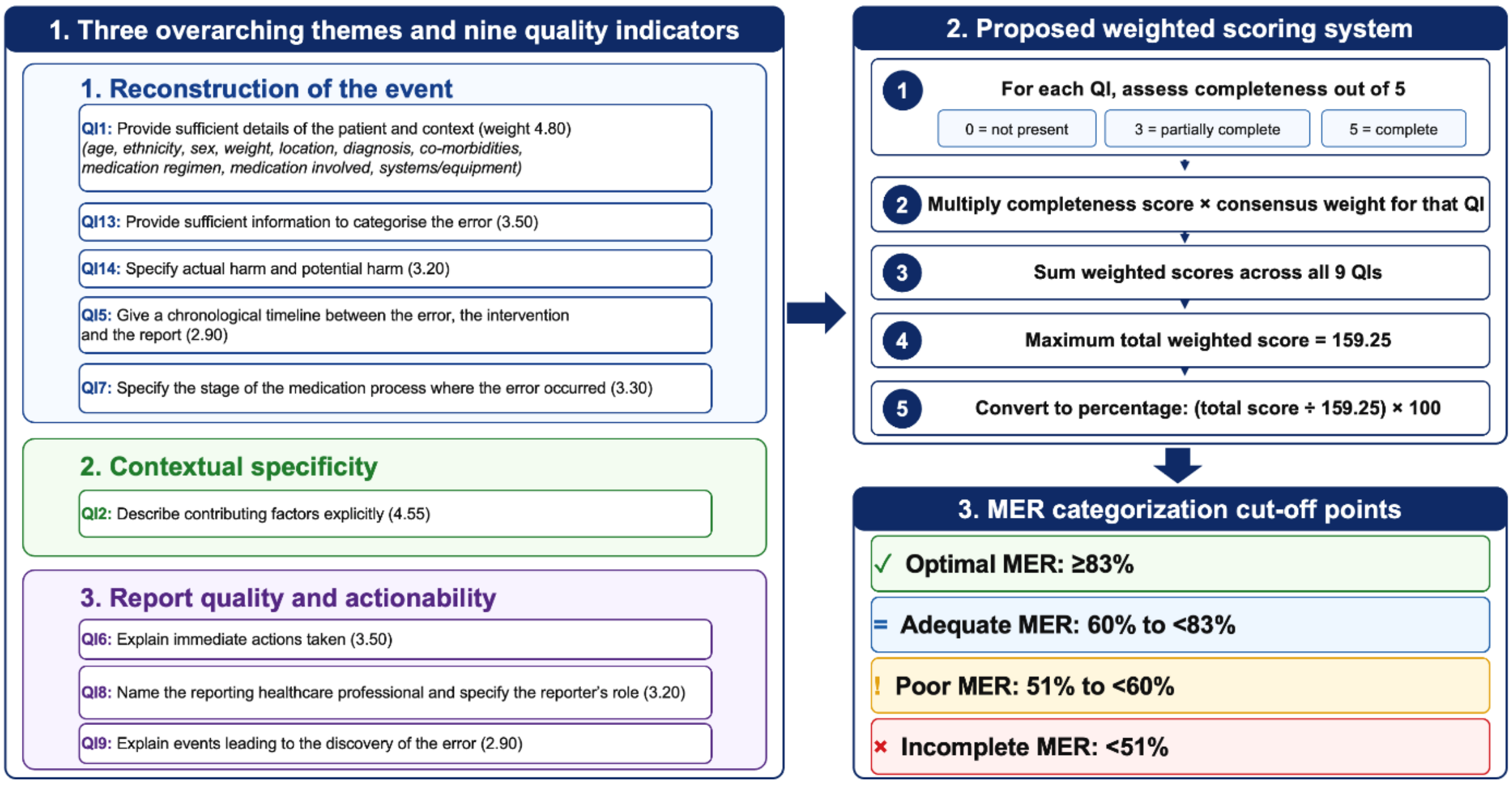

Figure 1: Conceptual figure of QoMER development

Minor comments
RC6: The abstract is generally clear and well structured; however, some wording still overstates the maturity and applicability of QoMER. More cautious language is recommended throughout.

AR6: Thank you for this comment. We have revised the abstract to better reflect QoMER's developmental stage. We now use more cautious terms, such as "consensus-derived preliminary framework" and "exploratory weighted scoring system," and clarify that the weights and cut-off points have not been validated. We also state that further validation, reliability testing, and real-world evaluation are needed before implementation.

RC7: The discussion section could be shortened slightly to reduce repetition and improve focus.

AR7: Thank you for this comment. The Discussion section has been shortened while maintaining the information.

RC8: The section discussing artificial intelligence and NLP applications is interesting but somewhat speculative relative to the scope of the current study. Consider shortening this section or framing it more cautiously as a future research direction.

AR8: Thank you for this comment. The discussion of artificial intelligence and natural language processing was somewhat speculative, given the scope of this consensus-based framework development study. We have therefore shortened this section and reframed it more cautiously as a potential future research direction.

Overall recommendation
This study addresses a meaningful gap in medication safety research and has clear potential relevance for improving the structure and evaluation of medication error reporting. However, substantial revisions are needed to better calibrate the conclusions, clarify the intended role of QoMER, and align the claims with the developmental nature of the current evidence.

VERSION 2 - REVIEW

**Reviewer**	**2**
**Name**	**Builes-Montaño, Carlos E.**
**Affiliation**	**Universidad de Antioquia, Internal Medicine**
**Date**	**06-Jul-2026**
**COI**	

Thank you for the opportunity to review the revised manuscript and the authors' responses.

The authors have addressed most of the major concerns raised in the previous review. In particular, the revised manuscript now provides a clearer conceptual definition of QoMER as a consensus-derived preliminary quality assessment framework rather than a validated measurement instrument. The authors have also moderated several claims regarding implementation readiness, clarified the exploratory nature of the weighted scoring system and cut-off thresholds, strengthened the limitations section, and added a conceptual figure to improve the presentation of the framework.

The revised manuscript is substantially improved and now more appropriately reflects the developmental nature of the study. The study design remains appropriate for the stated aim, the research question is clear, the methods are generally reproducible, and the results address the objective of developing a consensus-derived framework for assessing the quality of medication error reports.

I have only one remaining minor concern. Some wording in the Discussion and Conclusion still appears slightly stronger than warranted by the evidence generated in this study, particularly statements implying potential transformation of reporting systems or improvement of medication safety outcomes. I recommend that the authors make one final editorial adjustment to ensure that all implementation- and impact-oriented statements are clearly framed as future possibilities pending empirical validation, reliability testing, usability assessment, and real-world evaluation.

Overall, I consider the revised manuscript suitable for publication after minor editorial adjustment. The remaining issue relates only to wording and cautious framing of a few implementation-oriented statements, rather than to the study design, methods, results, or scientific validity. I do not think that an additional round of peer review is necessary. The final assessment of whether the wording has been sufficiently softened could be handled by the editor during the editorial revision process.

VERSION 2 - AUTHOR RESPONSE

Reviewer: 2
Dr. Carlos E. Builes-Montaño, Universidad de Antioquia, Hospital Pablo Tobón Uribe
Comments to the Author:
Thank you for the opportunity to review the revised manuscript and the authors' responses.

The authors have addressed most of the major concerns raised in the previous review. In particular, the revised manuscript now provides a clearer conceptual definition of QoMER as a consensus-derived preliminary quality assessment framework rather than a validated measurement instrument. The authors have also moderated several claims regarding implementation readiness, clarified the exploratory nature of the weighted scoring system and cut-off thresholds, strengthened the limitations section, and added a conceptual figure to improve the presentation of the framework.

The revised manuscript is substantially improved and now more appropriately reflects the developmental nature of the study. The study design remains appropriate for the stated aim, the research question is clear, the methods are generally reproducible, and the results address the objective of developing a consensus-derived framework for assessing the quality of medication error reports.

I have only one remaining minor concern. Some wording in the Discussion and Conclusion still appears slightly stronger than warranted by the evidence generated in this study, particularly statements implying potential transformation of reporting systems or improvement of medication safety outcomes. I recommend that the authors make one final editorial adjustment to ensure that all implementation- and impact-oriented statements are clearly framed as future possibilities pending empirical validation, reliability testing, usability assessment, and real-world evaluation.

Overall, I consider the revised manuscript suitable for publication after minor editorial adjustment. The remaining issue relates only to wording and cautious framing of a few implementation-oriented statements, rather than to the study design, methods, results, or scientific validity. I do not think that an additional round of peer review is necessary. The final assessment of whether the wording has been sufficiently softened could be handled by the editor during the editorial revision process.

Reviewer: 2
If you have selected 'Yes' above, please provide details of any competing interests.: Not applicable

---
