# Supplementary material for: Developing Quality of Medication Error Reports (QoMER): A Consensus-Based Preliminary Framework Using a Modified Nominal Group Technique in the UK
Source: BMJ Open. 2026 Sep 2;16(9):e115872. doi: 10.1136/bmjopen-2025-115872 (PMC13548226; doi:10.1136/bmjopen-2025-115872)
Supplement: Supplementary file 3 [file bmjopen-16-9-s001.docx]

# Title

Developing QoMER—Quality of Medication Error Reports: A Consensus-Based Scoring Tool Using a Modified Nominal Group Technique in the UK

# Supplementary A

Round-robin format recording of question no. 1: 3 rounds were repeated, generating 32 ideas, which are listed below.

1. Impact on patients/staff
2. Number of 10-fold dosage errors
3. Age of patient, demographics
4. Location of error
5. Medicines involved
6. As soon as possible
7. where the medication process error occurred
8. Can the reporter describe the root cause of the error?
9. Type and severity of the reaction to the drug.
10. Description of the severity of the incident
11. Location and time of the incident
12. Does the report allow adequate feedback to the individuals involved?
13. Number of 10-fold dosage errors reaching the patient
14. Patient details, including e.g. age, gender, ethnicity, consider not including other mandatory fields in our trust reporting system, such as Mental Health Act (MHA) section or Care Programme Approach (CPA) status, may put people off having to add in unnecessary information
15. If any systems or equipment were involved (e.g. pump/Electronic Prescribing System)
16. Who administered the drug, and what was the route of administration?
17. The Main condition and other co-morbidities
18. Who made the error - what is their competence/confidence, have they got experience with this drug?
19. What were the recognised error-inducing factors (fatigue, interruption, ambiguity) involved?
20. Is the report descriptive only, or does it allow for reflection/self-analysis?
21. Which of the 5 Rs was the reason for the medication error
22. At what stage of the process did the error occur? Prescribing, supply, administration?
23. If you have the report, you can formulate a way to reduce the future risk of the error recurring, and ultimately the risk of patient harm.
24. What can we do to stop the error from happening again?
25. Detailed facts of what actually happened & what action was taken. A reporting system that encourages / prompts consideration of influencing factors.
26. any indication that an appropriate level of duty of candour
27. How quickly/easily was the error recognised (time delay may indicate a knowledge issue or a supervision issue) if it was recognised at the time? How it was recognised, and whether anything influenced the recognition of the error (e.g. presence of a supervisor or presence of a family member)
28. Does the report account for any communication barriers between the patient and the prescriber?
29. What aspect of the system do you think could have contributed to the error in as much detail as specified in the guidance, with some tick box and scoring - e.g. lack of specific training, shortage of staff, pack appearance?
30. Nature/category of the error
31. presence of an unusual context surrounding the error
32. Reporters’ judgment of the complexity of the scenario within which the error occurred (maybe they provide a clinical complexity score).
