# Supplementary material for: Developing Quality of Medication Error Reports (QoMER): A Consensus-Based Preliminary Framework Using a Modified Nominal Group Technique in the UK
Source: BMJ Open. 2026 Sep 2;16(9):e115872. doi: 10.1136/bmjopen-2025-115872 (PMC13548226; doi:10.1136/bmjopen-2025-115872)
Supplement: Supplementary file 4 [file bmjopen-16-9-s002.docx]

# Title

Developing QoMER—Quality of Medication Error Reports: A Consensus-Based Scoring Tool Using a Modified Nominal Group Technique in the UK

# Supplementary B

During the discussion and clarification stage of question no. 1, participants included 13 quality indicators. The ideas that received fewer than seven votes were excluded, as shown below.

| Quality indicators | Inclusion votes |
| --- | --- |
| - Sufficient details of the patient and context:(diagnosis, co-morbidities, medication regimen, medication involved in the incident, systems or equipment involved (e.g. pump/electronic prescribing system | 11 |
| - Contributing factors | 10 |
| - Does the report contain sufficient information to categorise the error? | 10 |
| - Categorise – 5Rs | 5 |
| - Does the report contain the circumstances of the error? (Communication barriers) | 11 |
| - How is the error discovered? | 7 |
| - Does the report give a chronological timeline? | 10 |
| - Is it self-reported or not? What is the role of the reporter? | 9 |
| - What are the immediate actions taken? | 9 |
| - What preventive future actions would you recommend? | 6 |
| - Does the report allow adequate feedback to the individuals involved? | 3 |
| - Specify the actual harm and the potential harm | 11 |
| - Stage of the medication process | 8 |
