# Supplementary material for: Developing Quality of Medication Error Reports (QoMER): A Consensus-Based Preliminary Framework Using a Modified Nominal Group Technique in the UK
Source: BMJ Open. 2026 Sep 2;16(9):e115872. doi: 10.1136/bmjopen-2025-115872 (PMC13548226; doi:10.1136/bmjopen-2025-115872)
Supplement: Supplementary file 5 [file bmjopen-16-9-s003.docx]

# Title

Developing QoMER—Quality of Medication Error Reports: A Consensus-Based Scoring Tool Using a Modified Nominal Group Technique in the UK

# Supplementary C

**During the round-robin format recording stage of the second question,** one round generated 11 ideas for the scoring systems presented below.

1. 5-point Likert scale
2. Could weight each indicator's contribution to the total based on its importance.
3. When rating, I would use a scoring system with 1 for an included indicator and 0 for an excluded indicator.
4. Weighted yes/no
5. Weighted yes/no
6. Likert scale
7. Weighted yes/no
8. Weighted Likert scale
9. Weighted binary system
10. Weighted yes/no
11. Likert scale using not at all, partially, fully
